# Supplementary material for: A multi-task convolutional deep neural network for variant calling in single molecule sequencing
Source: Nat Commun. 2019 Mar 1;10:998. doi: 10.1038/s41467-019-09025-z (PMC6397153; doi:10.1038/s41467-019-09025-z)
Supplement: Supplementary file 1 — Supplementary Information [file 41467_2019_9025_MOESM1_ESM.pdf]

Clairvoyante: a multi-task convolutional deep neural network for variant calling in Single Molecule Sequencing – Supplementary Materials

Ruibang Luo, Fritz J. Sedlazeck, Tak-Wah Lam, Michael C. Schatz

Supplementary Figures 3
Supplementary Figure 1. IGV screen capture of the novel variants. 7
Supplementary Figure 2. Network Visualization. 11
Supplementary Figure 3. Summary of the reason for variant calling failure. 12
Supplementary Tables 13
Supplementary Table 1. Performance of using Clairvoyante for variant calling genome-wide on Illumina, PacBio and ONT datasets. 13
Supplementary Table 2. Summary of the best precision, recall, and F1-score of the benchmarks at common variant sites and on whole-genome. 14
Supplementary Table 3. Additional benchmarks. 15
Supplementary Table 4. Reasons for false positive variant calls. 16
Supplementary Table 5. Reasons for false negative variant calls. 19
Supplementary Table 6. Performance of variant calls in HG003 on three different models 22
Supplementary Table 7. F1-scores of different datasets on different network designs. 23
Supplementary Data 24
Supplementary Data 1: 3,135 potential novel variants. 24
Supplementary Note 25
Unit tests 25
Characterization of false positives and false negatives 25
Can lower learning rate and longer training provide better performance? 26
Can a model train on truth variants from multiple samples provide better performance? 26
Can a higher input data quality improve the variant calling performance? 26
Network topology and capacity evaluation 27
Pseudo code for generating the input 28
Data Sources 29
Truth Variants (Genome in a Bottle dataset version 3.3.2) 29

Common Variants (1000 genome project, phase 3) 29
Illumina Data 29
Pacific Bioscience (PacBio) Data 30
Oxford Nanopore (ONT) Data 30
Call Variants at Common Variant Sites 31
Commands 31
Benchmarking 34
Commands 34
Call Variants Genome-wide 35
Commands 35
Supplemental References 37

Supplementary Figures

a) 1-566371-A-G-0/1

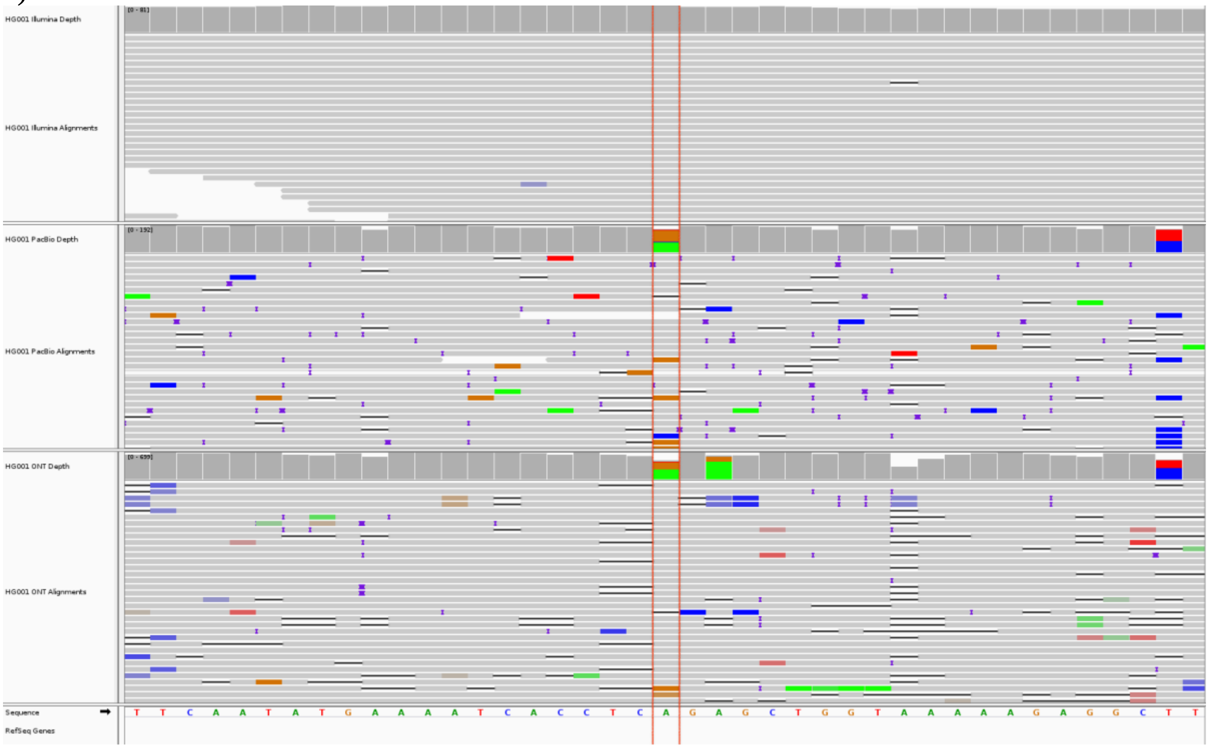

b) X-143214235-G-T-0/1

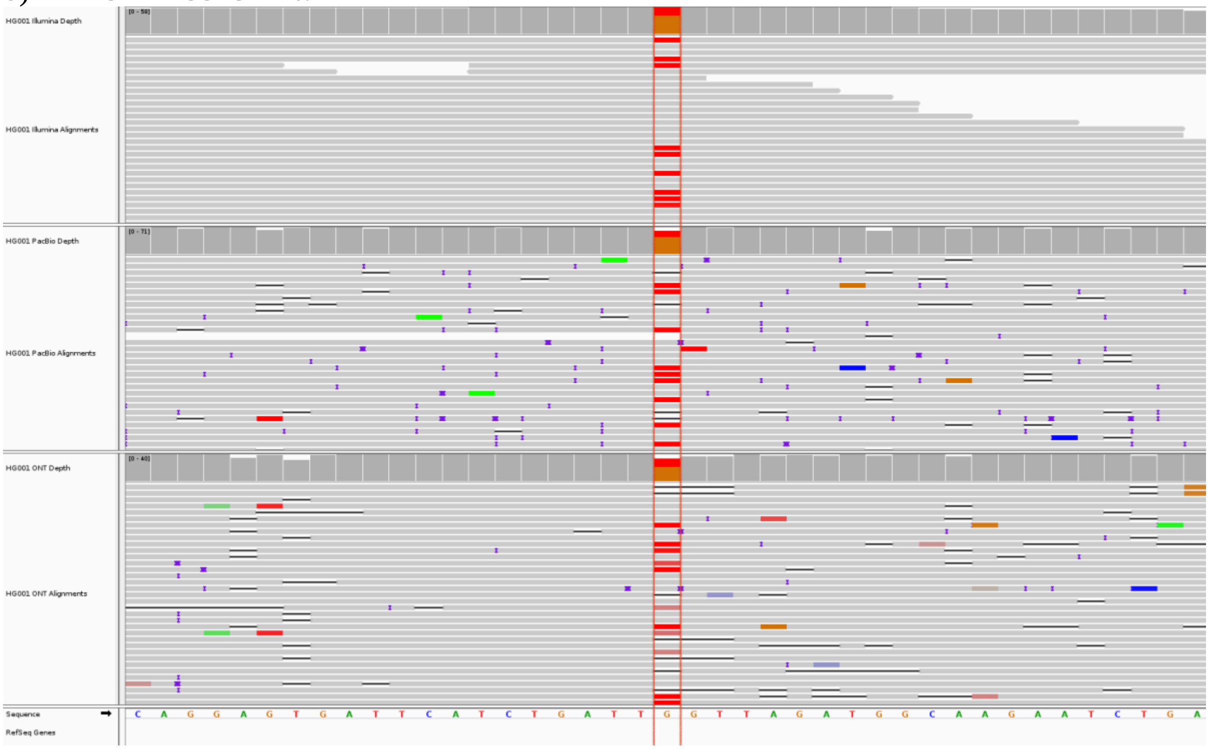

c) 13-104270904-TA-T-1/1

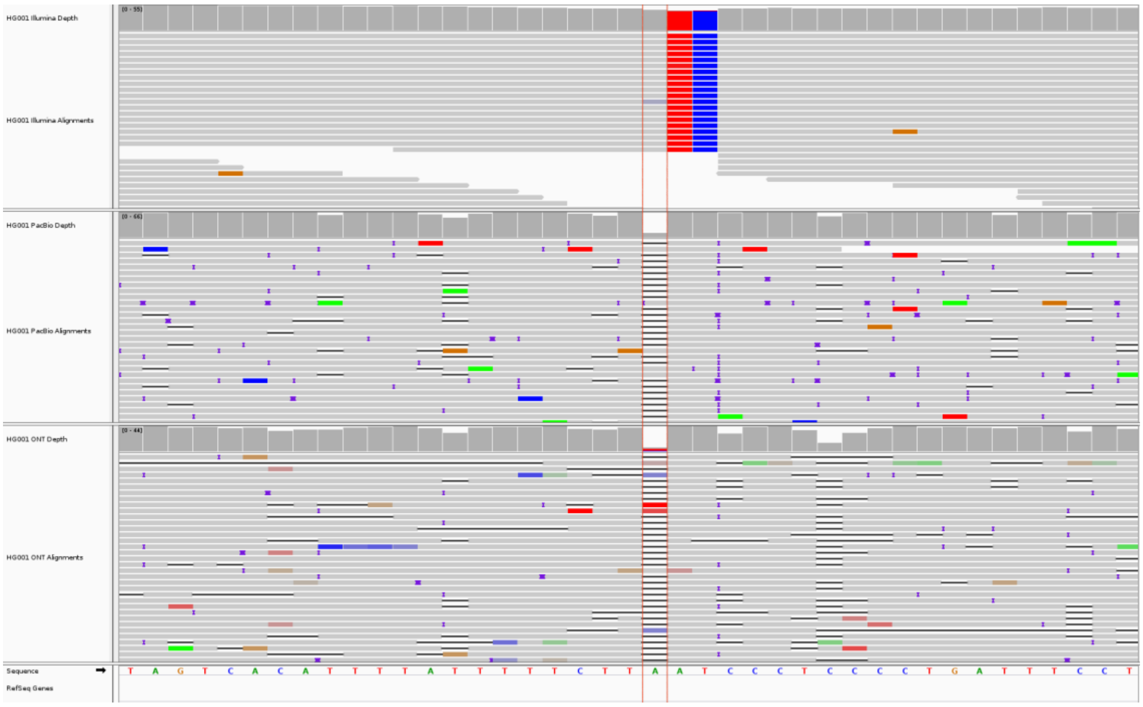

d) X-140640513-T-G-0/1

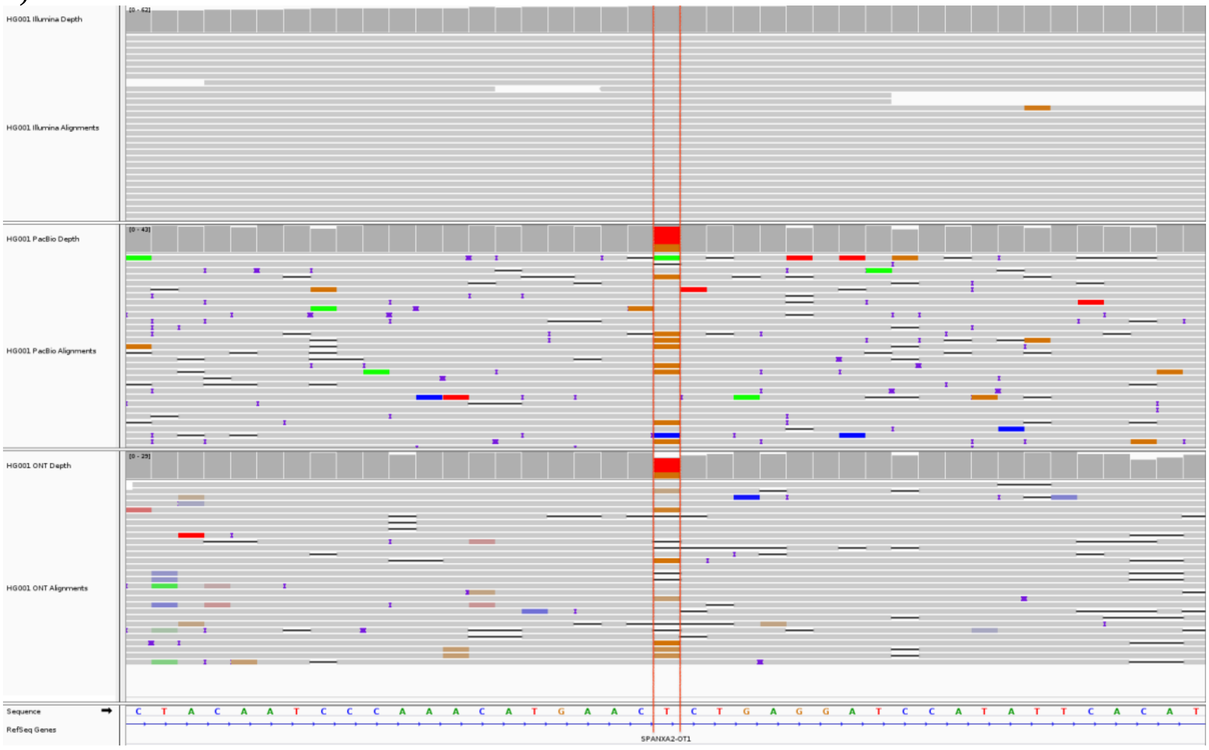

e) X-143218136-G-T-0/1

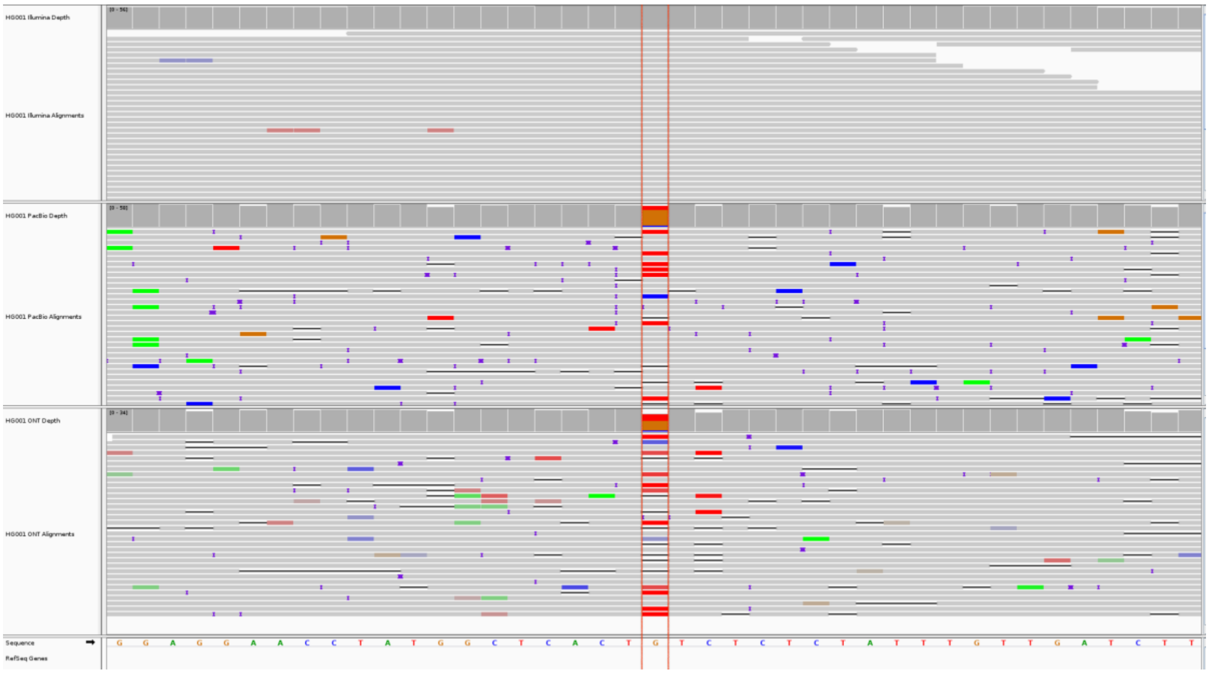

f) 9-113964088-G-T-0/1

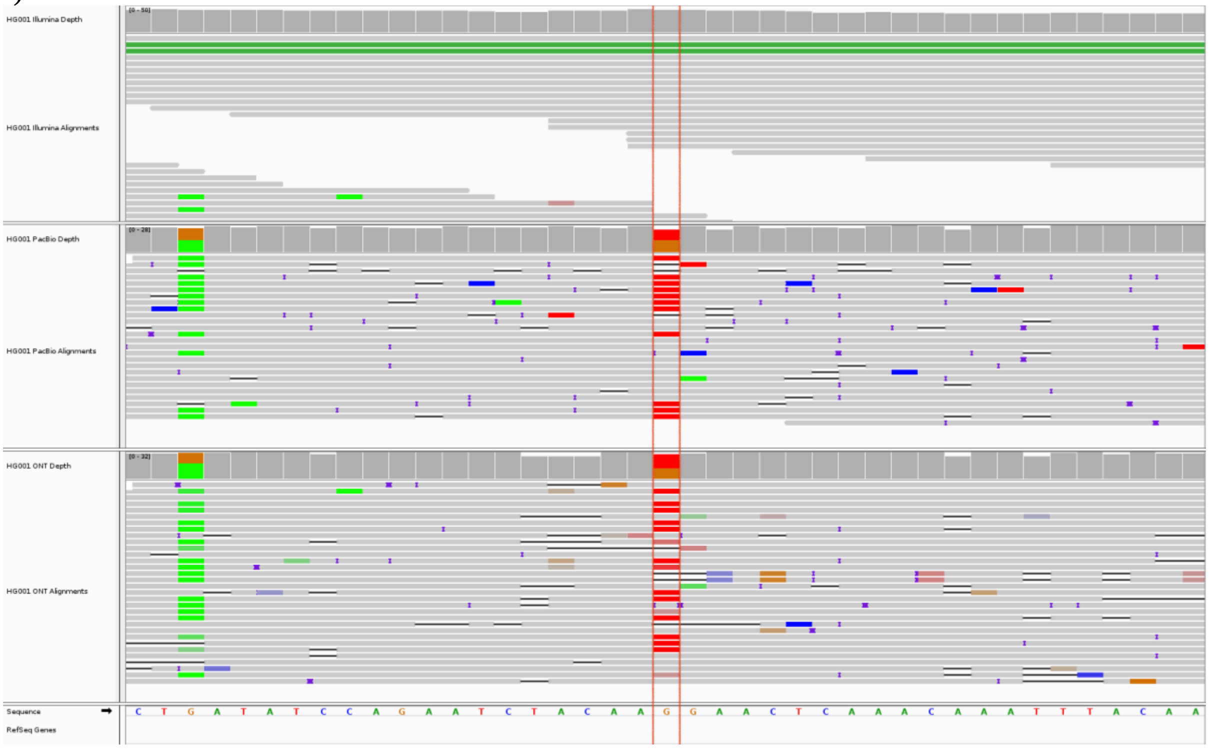

g) 7-89312043-G-T-0/1

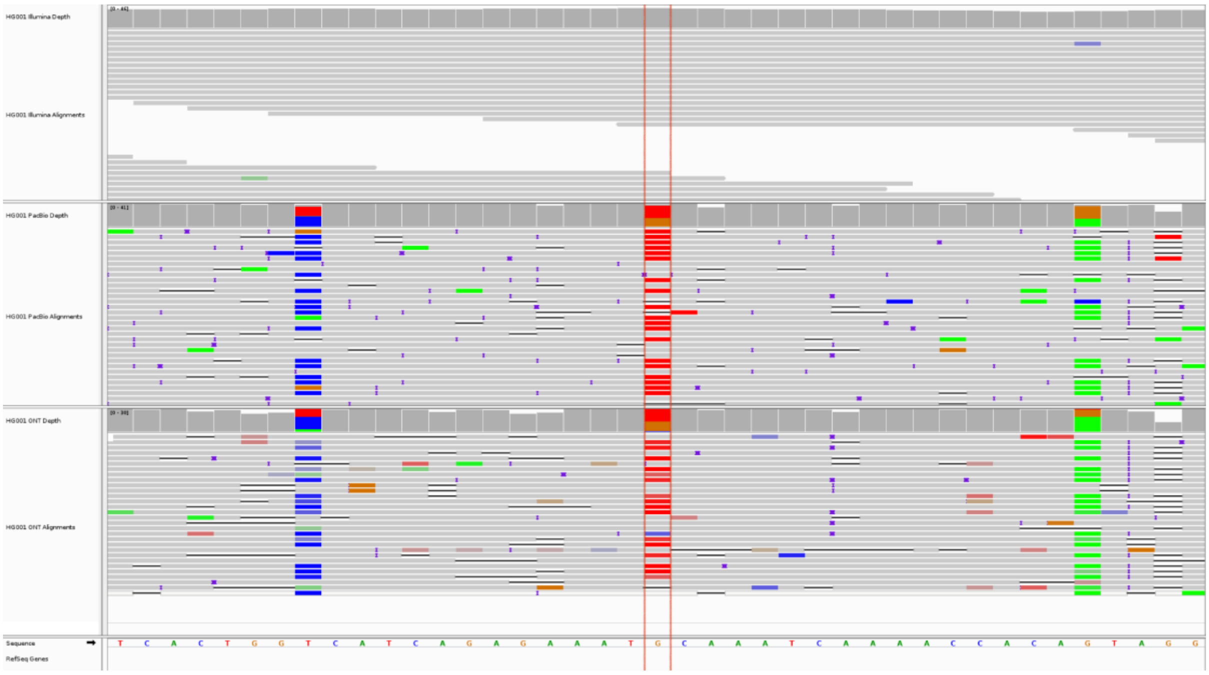

h) 2-163811179-T-G-0/1

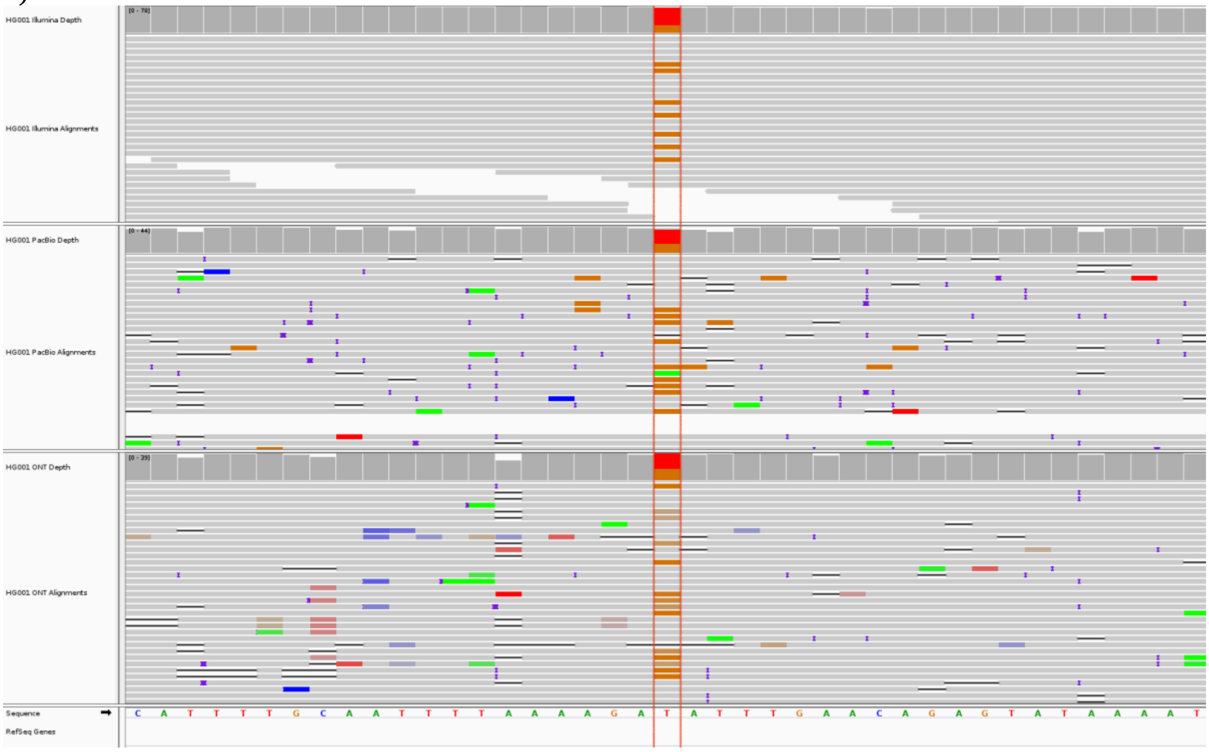

i) 10-65260789-C-T-1/1

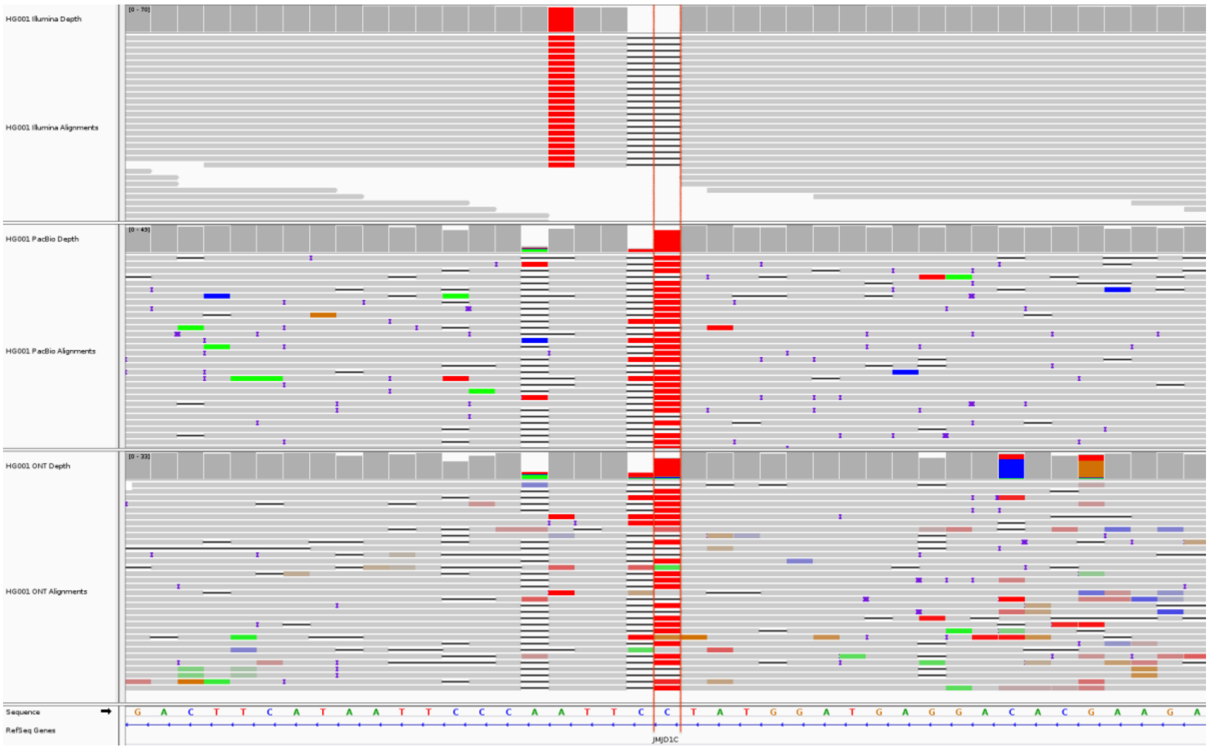

Supplementary Figure 1. IGV screen capture of the novel variants.

The IGV screen capture of 9 variants (from a to i, second to tenth in the top 10 in variant quality) identified by PacBio and ONT in HG001 but are not yet indexed in GIAB's truth variant dataset.

a) input

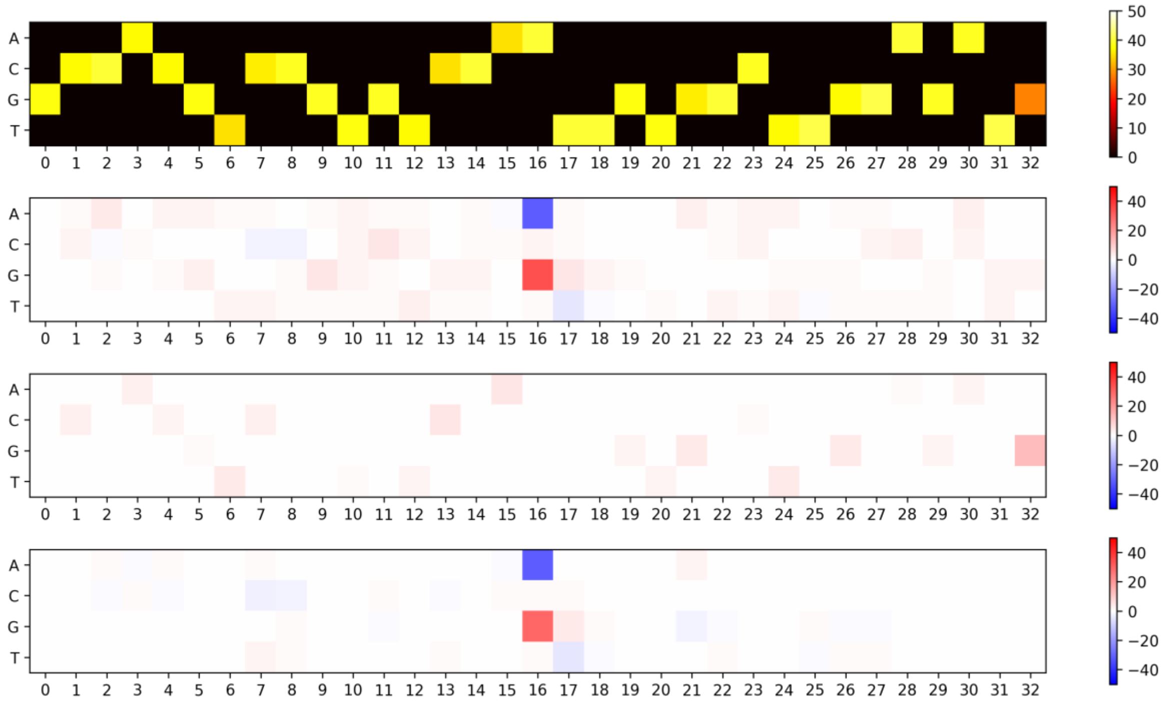

b) conv1

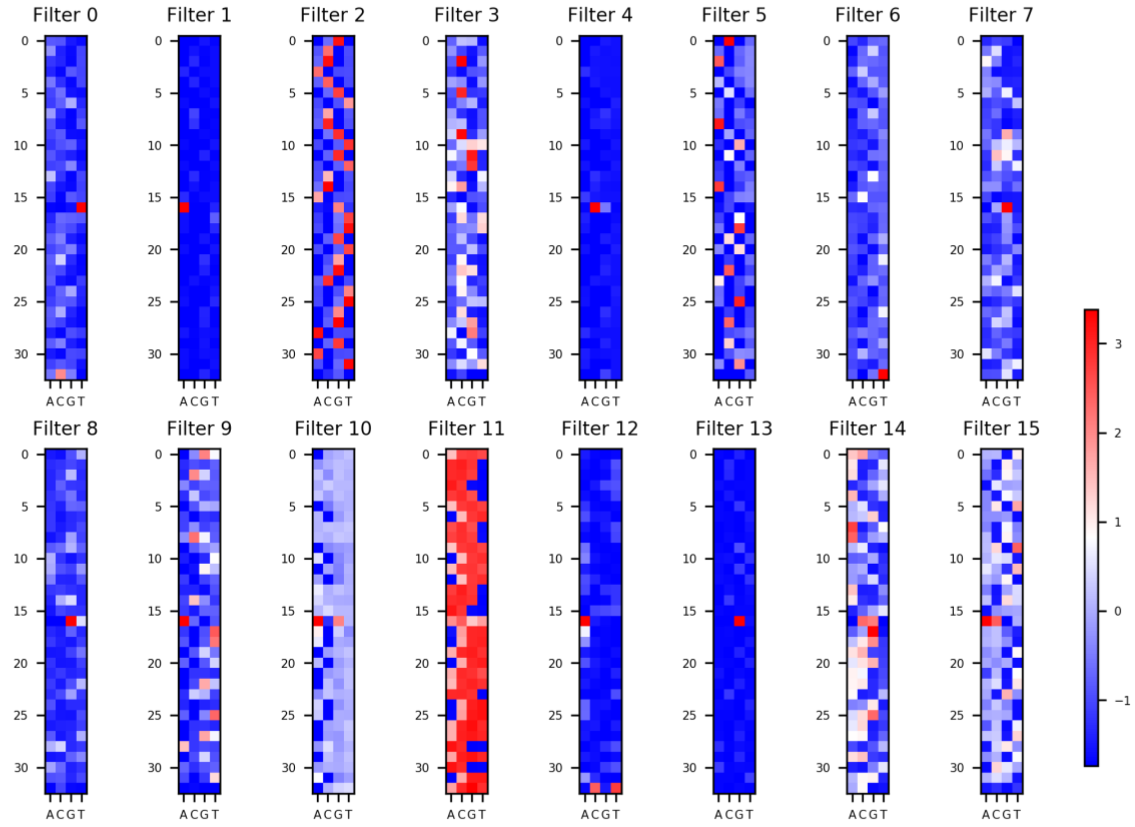

c) conv2

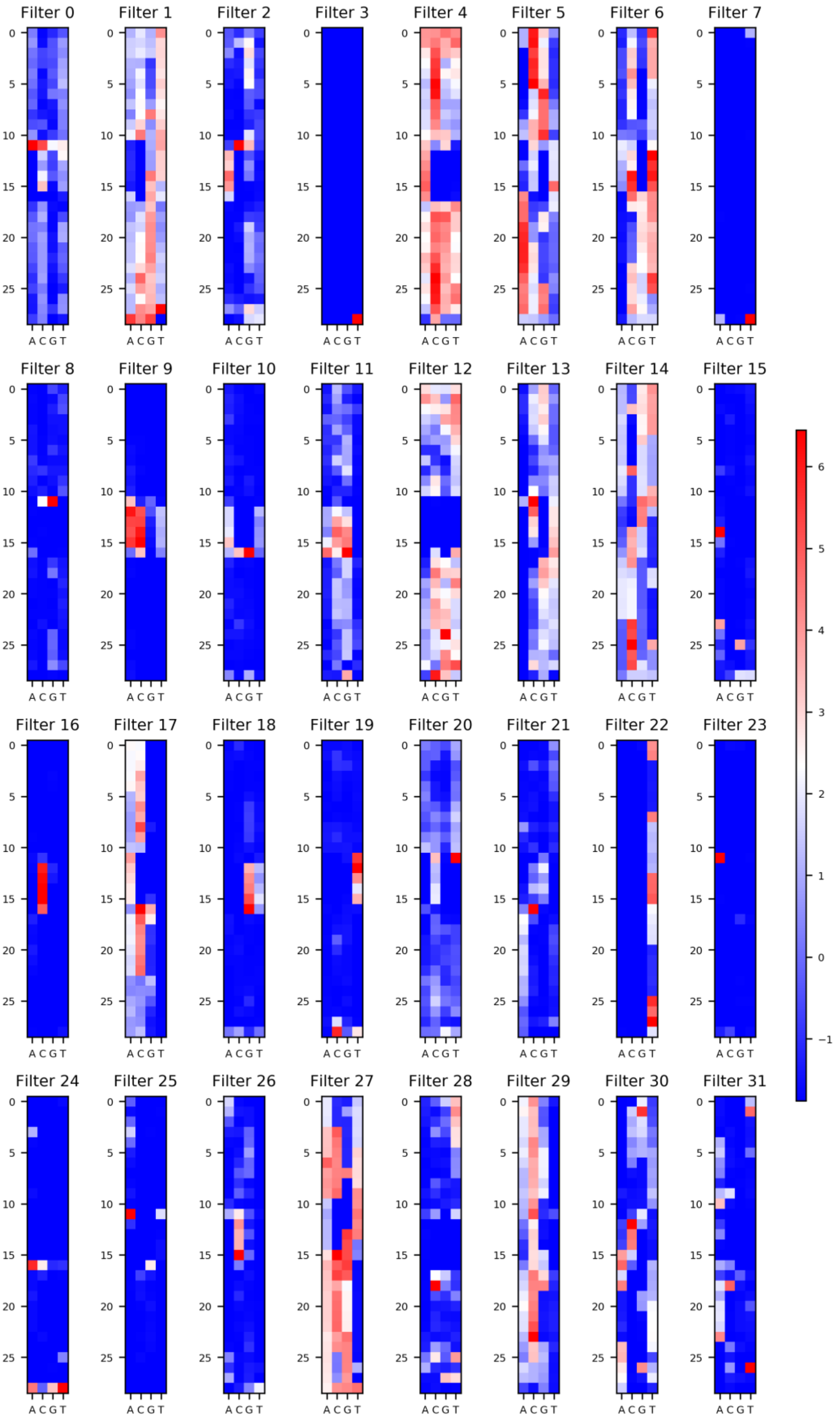

Filter 0
Filter 1
Filter 2
Filter 3
Filter 4
Filter 5
Filter 6
Filter 7
Filter 8
Filter 9
Filter 10
Filter 11
Filter 12
Filter 13
Filter 14
Filter 15
Filter 16
Filter 17
Filter 18
Filter 19
Filter 20
Filter 21
Filter 22
Filter 23
Filter 24
Filter 25
Filter 26
Filter 27
Filter 28
Filter 29
Filter 30
Filter 31
A C G T
0
5
10
15
20
25
6
5
4
3
2
1
0
−1

d) conv3

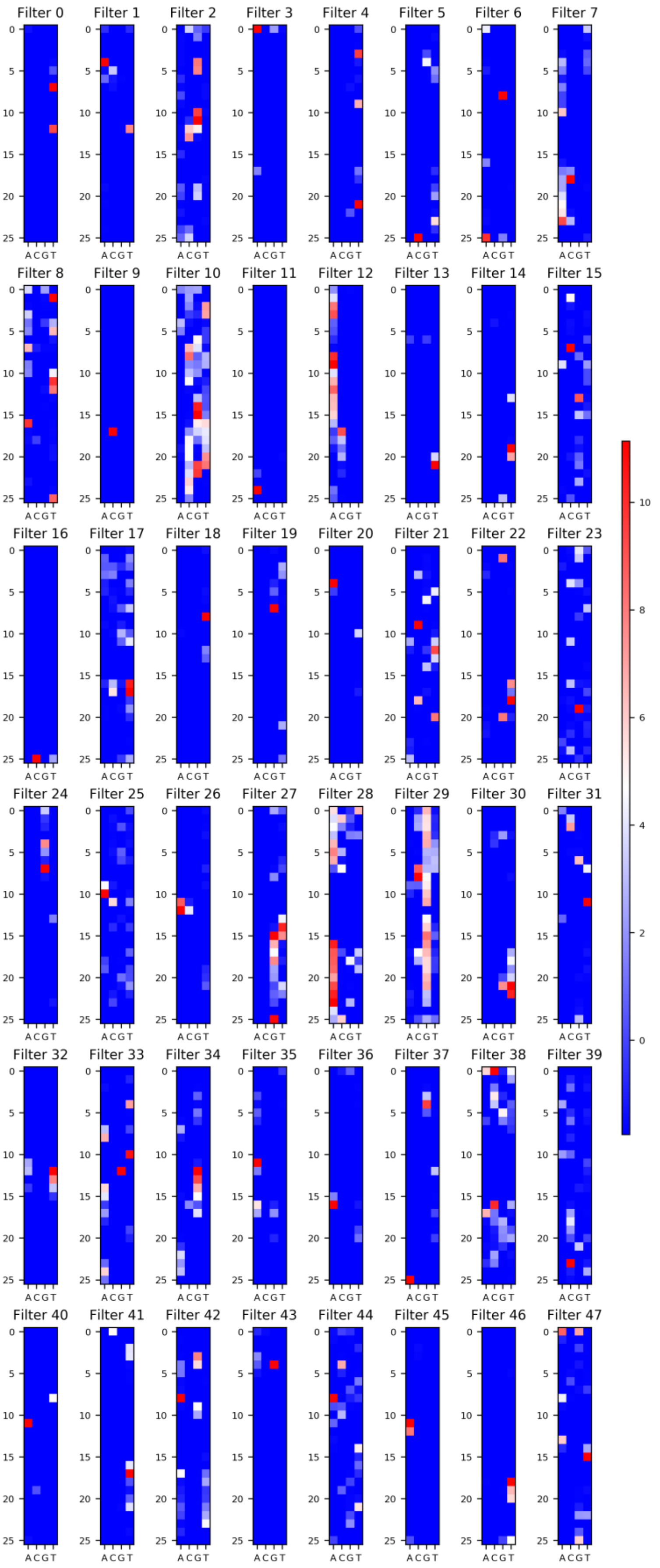
Filter 0
Filter 1
Filter 2
Filter 3
Filter 4
Filter 5
Filter 6
Filter 7
Filter 8
Filter 9
Filter 10
Filter 11
Filter 12
Filter 13
Filter 14
Filter 15
Filter 16
Filter 17
Filter 18
Filter 19
Filter 20
Filter 21
Filter 22
Filter 23
Filter 24
Filter 25
Filter 26
Filter 27
Filter 28
Filter 29
Filter 30
Filter 31
Filter 32
Filter 33
Filter 34
Filter 35
Filter 36
Filter 37
Filter 38
Filter 39
Filter 40
Filter 41
Filter 42
Filter 43
Filter 44
Filter 45
Filter 46
Filter 47
0
5
10
15
20
25
A C G T
0
2
4
6
8
10

e) fc4

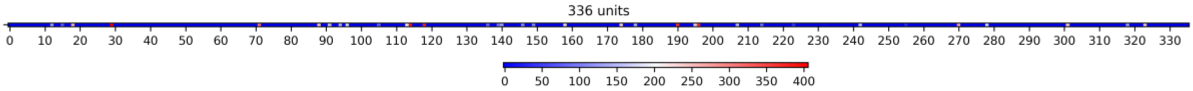

f) fc5

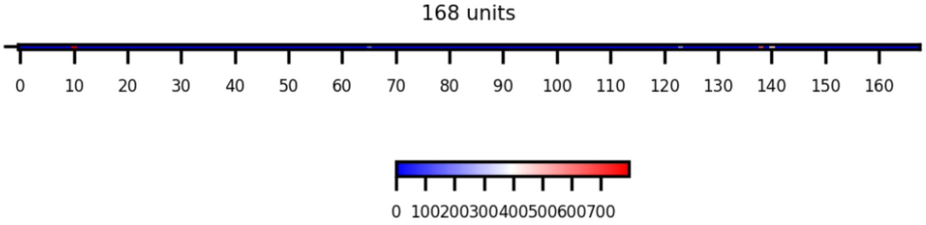

g) output

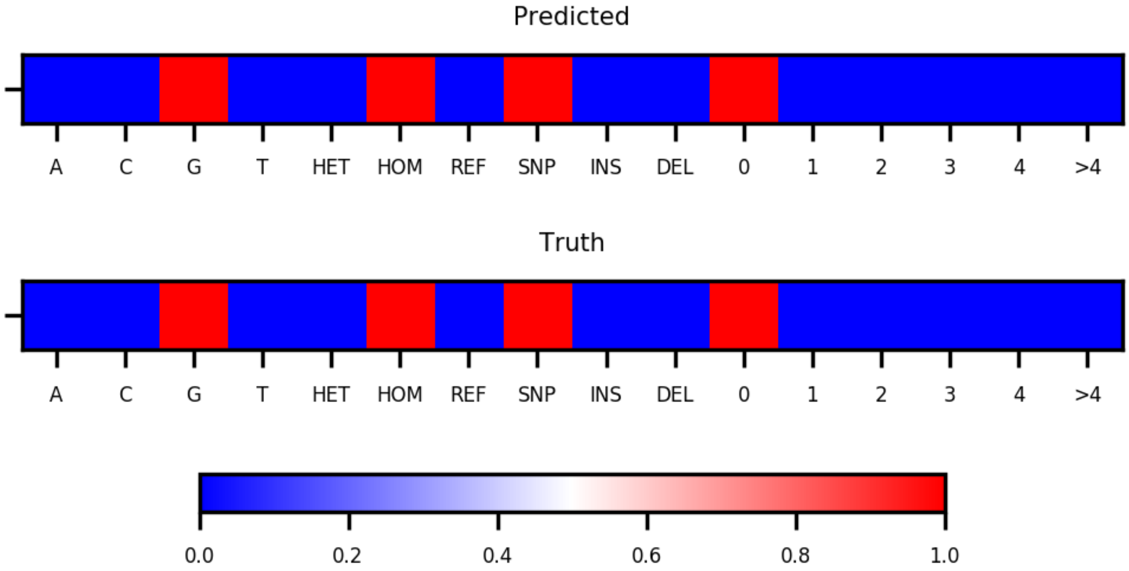

Supplementary Figure 2. Network Visualization.

Input and node activations in all hidden layers and output layers of an A>G SNP variant. All plots use sample HG002 test against a model trained with HG001 samples for a thousand epochs at 1e^{-3} learning rate. a) input; b) conv1; c) conv2; d) conv3; e) fc4; f) fc5; g) output.

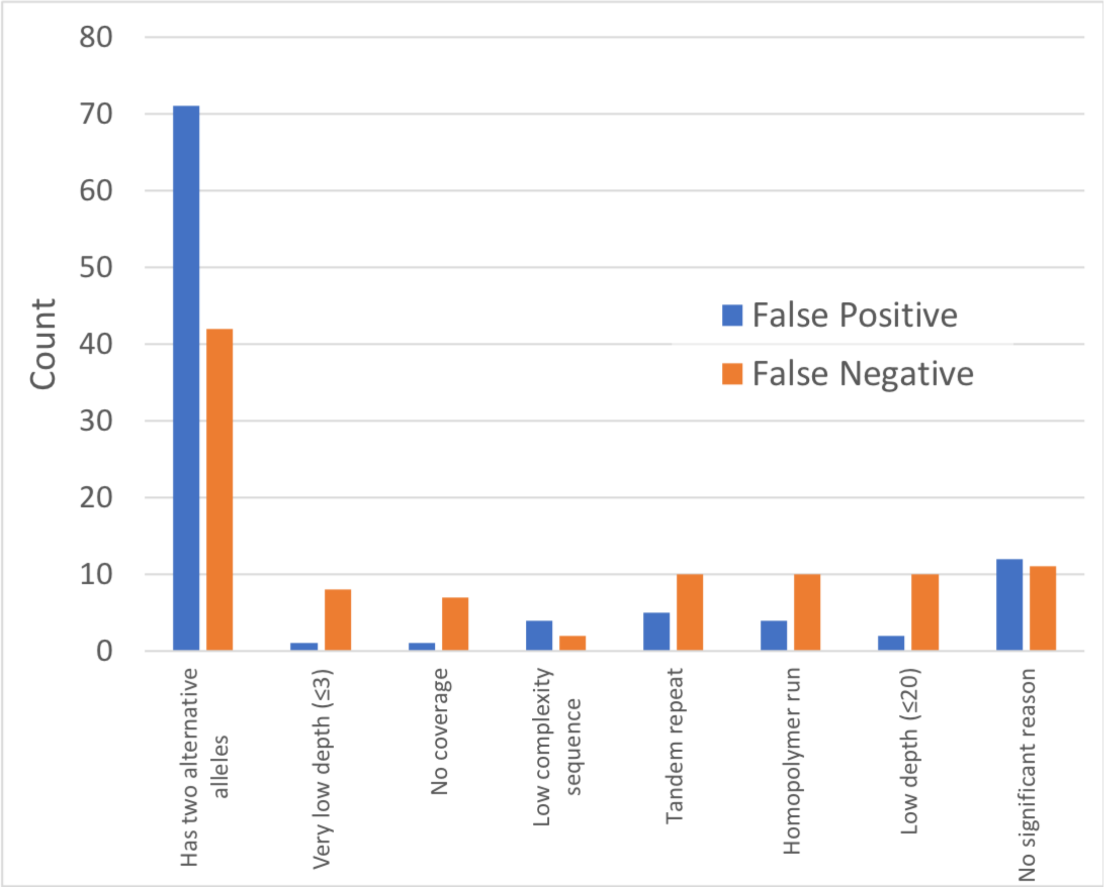

Supplementary Figure 3. Summary of the reason for variant calling failure.

Summary of the reason of failure on 100 randomly picked false positive variants, and 100 randomly picked false negative variants.

Supplementary Tables

Supplementary Table 1. Performance of using Clairvoyante for variant calling genome-wide on Illumina, PacBio and ONT datasets.

All models were trained for 1000 epochs with learning rate at $1e^{-3}$.

Seq. Tech	Train using Variants in	Call Variants in	Alt. Allele Freq. Cutoff	Best Variant Quality Cutoff	Time Consumption	Overall			SNP			Indel		
						Precision	Recall	F1 Score	Precision	Recall	F1 Score	Precision	Recall	F1 Score
Illumina	HG001	HG001	0.1	189	1:08	98.16%	98.93%	98.55%	98.10%	99.92%	99.00%	98.63%	96.96%	97.79%
			0.2	182	0:43	98.41%	98.88%	98.65%	98.38%	99.90%	99.13%	98.70%	95.10%	96.86%
			0.25	180	0:26	98.71%	97.95%	98.33%	98.72%	99.82%	99.27%	98.62%	87.33%	92.63%
		HG002	0.1	192	1:11	98.13%	98.77%	98.45%	98.08%	99.81%	98.94%	98.50%	96.54%	97.51%
			0.2	183	0:41	98.35%	98.77%	98.56%	98.33%	99.78%	99.05%	98.51%	94.90%	96.67%
			0.25	182	0:30	98.67%	97.88%	98.27%	98.70%	99.69%	99.19%	98.43%	87.34%	92.55%
	HG002	HG001	0.1	198	1:16	98.59%	98.50%	98.54%	98.68%	99.88%	99.27%	97.98%	93.37%	95.62%
			0.2	192	0:47	98.75%	98.39%	98.57%	98.84%	99.86%	99.35%	98.07%	91.69%	94.78%
			0.25	184	0:25	98.94%	97.60%	98.27%	99.07%	99.78%	99.42%	97.91%	85.01%	91.00%
		HG002	0.1	195	1:07	98.53%	98.59%	98.56%	98.59%	99.85%	99.22%	98.12%	93.78%	95.90%
			0.2	188	0:44	98.71%	98.50%	98.61%	98.77%	99.81%	99.29%	98.22%	92.24%	95.14%
			0.25	182	0:25	98.95%	97.73%	98.33%	99.05%	99.71%	99.38%	98.11%	85.73%	91.50%
	GATK UnifiedGenotyper, HG001			51	0:46	99.43%	99.42%	99.43%	99.53%	99.91%	99.72%	98.76%	96.47%	97.60%
	GATK HaplotypeCaller, HG001			5	8:45	99.69%	99.83%	99.76%	99.77%	99.97%	99.87%	99.22%	98.97%	99.09%
	GATK UnifiedGenotyper, HG002			4	0:46	98.76%	99.41%	99.08%	98.73%	99.85%	99.29%	98.91%	96.57%	97.73%
	GATK HaplotypeCaller, HG002			5	8:23	99.59%	99.81%	99.70%	99.62%	99.90%	99.76%	99.39%	99.24%	99.32%
PacBio	HG001	HG001	0.1	157	9:46	96.31%	88.63%	92.31%	96.72%	99.49%	98.09%	79.19%	31.13%	44.69%
			0.2	130	3:53	98.12%	87.62%	92.57%	98.96%	96.60%	97.77%	75.87%	31.50%	44.52%
			0.25	125	2:01	98.62%	83.11%	90.20%	99.39%	91.38%	95.22%	78.55%	27.10%	40.30%
		HG002	0.1	153	9:24	97.00%	89.08%	92.87%	97.90%	99.15%	98.52%	71.57%	34.26%	46.34%
			0.2	132	3:34	97.93%	88.30%	92.86%	99.03%	97.05%	98.03%	73.37%	34.06%	46.53%
			0.25	116	1:46	98.06%	84.69%	90.89%	99.18%	92.53%	95.74%	75.56%	31.24%	44.21%
	HG002	HG001	0.1	163	14:55	95.58%	86.69%	90.92%	96.64%	98.96%	97.79%	59.19%	24.52%	34.67%
			0.2	147	3:29	97.49%	85.64%	91.18%	98.94%	96.13%	97.51%	58.24%	23.65%	33.64%
			0.25	139	1:39	98.16%	81.47%	89.04%	99.27%	90.90%	94.90%	66.31%	21.11%	32.02%
		HG002	0.1	150	15:31	97.10%	89.31%	93.04%	98.33%	99.14%	98.73%	69.19%	39.77%	50.51%
			0.2	134	3:34	98.09%	88.51%	93.05%	99.35%	97.30%	98.32%	72.20%	36.59%	48.57%
			0.25	118	1:46	98.20%	84.76%	90.98%	99.47%	92.77%	96.00%	72.94%	31.44%	43.94%
Oxford Nanopore (rel3)	HG001	HG001	0.1	140	13:01	86.24%	71.01%	77.89%	86.79%	91.85%	89.25%	55.36%	10.69%	17.93%
			0.2	139	4:47	87.24%	70.21%	77.80%	87.72%	87.97%	87.85%	59.05%	9.90%	16.96%
			0.25	136	2:40	87.76%	68.51%	76.95%	88.25%	82.86%	85.47%	59.41%	9.26%	16.03%
			0.35	130	1:30	90.96%	57.43%	70.41%	91.34%	65.82%	76.51%	67.35%	6.62%	12.06%
Oxford Nanopore (rel5)	HG001	HG001	0.2	162	5:53	88.76%	85.81%	87.26%	88.95%	93.76%	91.29%	72.10%	8.32%	14.92%
			0.25	159	3:12	89.14%	82.20%	85.53%	89.34%	90.09%	89.71%	72.45%	8.02%	14.45%
			0.35	148	1:51	91.22%	67.88%	77.83%	91.48%	75.18%	82.53%	71.25%	6.54%	11.98%

Supplementary Table 2. Summary of the best precision, recall, and F1-score of the benchmarks at common variant sites and on whole-genome.

Performance	Seq. Tech.	Genome	Best Precision		Best Recall		Best F1-score	
			Common Var.	Genome Wide	Common Var.	Genome Wide	Common Var.	Genome Wide
Overall	Illumina	HG001	99.75%	98.94%	99.59%	98.93%	99.67%	98.65%
		HG002	99.76%	98.95%	99.51%	98.59%	99.61%	98.61%
	PacBio	HG001	98.51%	98.62%	94.19%	88.63%	95.78%	92.57%
		HG002	98.07%	98.20%	94.30%	89.31%	96.03%	93.05%
	ONT	HG001	96.55%	90.96%	85.22%	71.01%	90.53%	77.89%
SNP	Illumina	HG001	99.93%	99.07%	99.92%	99.92%	99.92%	99.42%
		HG002	99.94%	99.05%	99.88%	99.85%	99.90%	99.38%
	PacBio	HG001	99.75%	99.39%	98.82%	99.49%	99.28%	98.09%
		HG002	99.59%	99.47%	99.01%	99.15%	99.30%	98.73%
	ONT	HG001	97.28%	91.34%	92.41%	91.85%	94.78%	89.25%
Indel	Illumina	HG001	98.30%	98.70%	96.89%	96.96%	97.58%	97.79%
		HG002	98.14%	98.51%	96.53%	96.54%	97.26%	97.51%
	PacBio	HG001	78.21%	79.19%	53.02%	31.50%	60.50%	44.69%
		HG002	77.70%	75.56%	53.13%	39.77%	62.52%	50.51%
	ONT	HG001	76.59%	67.35%	21.36%	10.69%	33.24%	17.93%

Supplementary Table 3. Additional benchmarks.

Additional benchmark performance of using Clairvoyante and other state-of-the-art variant callers.

Seq. Tech.	Tool	Model Trained on	Call Variants in	Excluding Indel >4bp	Best Variant Quality Cutoff	Time Consumption	Overall			SNP			Indel		
							Precision	Recall	F1 Score	Precision	Recall	F1 Score	Precision	Recall	F1 Score
Illumina	Vardict	N.A.	HG001		N.A.	2:51	90.56%	98.13%	94.19%	94.09%	99.46%	96.70%	70.64%	88.85%	78.71%
	LoFreq	N.A.	HG001		N.A.	4:03	SNP only			75.88%	94.24%	84.07%	-	-	-
	FreeBayes	N.A.	HG001		43	4:54	98.71%	97.95%	98.33%	98.72%	99.82%	99.27%	98.62%	87.33%	92.63%
	DeepVariant	TL + Chr1	HG001		3	9:43	99.94%	99.91%	99.93%	99.99%	99.96%	99.98%	99.64%	99.59%	99.62%
	FreeBayes	N.A.	Syndip		19	5:42	96.00%	94.21%	95.10%	98.91%	97.17%	98.03%	78.20%	74.54%	76.32%
	Clairvoyante	Syndip	Syndip		70	1:23	91.95%	93.05%	92.49%	93.49%	96.32%	94.88%	80.00%	71.13%	75.30%
	Clairvoyante	Syndip	Syndip	Y	70	1:23	92.75%	95.32%	94.02%	93.60%	96.22%	94.89%	84.76%	86.94%	85.83%
	Clairvoyante	Syndip	Syndip (Known)	Y	20	0:09	98.83%	98.22%	98.52%	99.23%	98.69%	98.96%	94.97%	93.82%	94.39%
	Clairvoyante	Syndip	HG001		76	1:05	92.17%	96.05%	94.07%	92.63%	97.71%	95.10%	89.08%	85.91%	87.46%
	Clairvoyante	Syndip	HG001	Y	76	1:05	92.77%	97.09%	94.88%	92.64%	97.72%	95.11%	93.71%	92.67%	93.18%
	Clairvoyante	Syndip	HG001 (Known)	Y	6	0:08	99.53%	99.50%	99.51%	99.81%	99.83%	99.82%	97.58%	97.16%	97.37%
	Clairvoyante	Syndip	HG002		74	1:11	92.19%	96.07%	94.09%	92.61%	97.55%	95.02%	89.25%	86.64%	87.93%
	Clairvoyante	Syndip	HG002	Y	74	1:11	92.73%	97.06%	94.85%	92.62%	97.56%	95.02%	93.63%	93.43%	93.53%
	Clairvoyante	Syndip	HG002 (Known)	Y	9	0:08	99.51%	99.43%	99.47%	99.77%	99.74%	99.75%	97.64%	97.13%	97.38%
	Clairvoyante	HG001	HG001, AF≥0.2	Y	182	0:43	98.41%	98.88%	98.65%	98.38%	99.90%	99.13%	98.70%	95.10%	96.86%
PacBio	Clairvoyante	HG001	HG001, AF≥0.2	Y	130	3:53	98.12%	87.62%	92.57%	98.96%	96.60%	97.77%	75.87%	31.50%	44.52%
Oxford Nanopore	Vardict	N.A.	HG001		N.A.	17:12	0.42%	0.01%	0.01%	2.56%	0.01%	0.01%	0.04%	0.00%	0.01%
	LoFreq	N.A.	HG001		N.A.	6:58	SNP only			82.69%	54.75%	65.88%	-	-	-
	DeepVariant	TL + Chr1	HG001 (Chr22)	Y	3	9:19	90.97%	66.77%	77.02%	91.61%	76.70%	83.50%	65.54%	8.12%	14.45%
	DeepVariant	TL + Chr21	HG001 (Chr1)	Y	3	50:14	93.47%	74.62%	82.99%	94.36%	84.39%	89.10%	65.68%	12.06%	20.38%
	DeepVariant	TL + Chr21	HG001 (Chr22)	Y	3	9:11	92.19%	67.34%	77.83%	92.87%	77.06%	84.23%	69.87%	10.33%	17.99%
	Clairvoyante	HG001 (Chr1)	HG001 (Chr22)	Y	174	0:06	88.99%	86.24%	87.59%	90.32%	93.79%	92.02%	72.54%	9.31%	16.50%
	Clairvoyante	HG001 (Chr21)	HG001 (Chr1)	Y	171	0:31	91.13%	89.82%	90.47%	91.32%	95.10%	93.17%	74.72%	13.14%	22.35%
	Clairvoyante	HG001 (Chr21)	HG001 (Chr22)	Y	171	0:06	89.95%	86.21%	88.04%	90.10%	93.92%	91.97%	72.71%	9.44%	16.71%
	Clairvoyante	HG001	HG001, AF≥0.2	Y	162	5:53	88.76%	85.81%	87.26%	88.95%	93.76%	91.29%	72.10%	8.32%	14.92%

Supplementary Table 4. Reasons for false positive variant calls.

The details of the 100 randomly picked false positive variant calls.

Chromosome	Position	Two alternative alleles	Low complexity	Tandem repeat	Polymer run	Low depth	Very low depth	No depth	No significant reason	Comments
10	14039455	1	0	0	0	0	0	0	0	
1	16087311	1	0	0	0	0	0	0	0	
1	59493096	1	0	0	0	0	0	0	0	
1	91826744	1	0	0	0	0	0	0	0	
1	94166813	1	0	0	0	0	0	0	0	
1	106818140	1	0	0	0	0	0	0	0	
1	112713064	1	0	0	0	0	0	0	0	
1	237731891	1	0	0	0	0	0	0	0	
2	53128510	1	0	0	0	0	0	0	0	
2	153914483	1	0	0	0	0	0	0	0	
3	15906230	1	0	0	0	0	0	0	0	
3	52533778	1	0	0	0	0	0	0	0	
3	64662932	1	0	0	0	0	0	0	0	
3	77235355	1	0	0	0	0	0	0	0	
3	140444430	1	0	0	0	0	0	0	0	
3	158722881	1	0	0	0	0	0	0	0	
3	197588041	1	0	0	0	0	0	0	0	
4	123431384	1	0	0	0	0	0	0	0	
4	178471865	1	0	0	0	0	0	0	0	
4	188319366	1	0	0	0	0	0	0	0	
5	19327541	1	0	0	0	0	0	0	0	
5	115414042	1	0	0	0	0	0	0	0	
5	158845705	1	0	0	0	0	0	0	0	
5	164452963	1	0	0	0	0	0	0	0	
6	21297653	1	0	0	0	0	0	0	0	
6	48723442	1	0	0	0	0	0	0	0	
6	114872467	1	0	0	0	0	0	0	0	
7	9630356	1	0	0	0	0	0	0	0	
7	14796687	1	0	0	0	0	0	0	0	
7	31449505	1	0	0	0	0	0	0	0	
7	70691874	1	0	0	0	0	0	0	0	
7	122427121	1	0	0	0	0	0	0	0	
8	23627961	1	0	0	0	0	0	0	0	
8	28234178	1	0	0	0	0	0	0	0	
8	50464952	1	0	0	0	0	0	0	0	
8	54576043	1	0	0	0	0	0	0	0	
8	87804634	1	0	0	0	0	0	0	0	

8	92126409	1	0	0	0	0	0	0	0	
9	83989667	1	0	0	0	0	0	0	0	
9	87327413	1	0	0	0	0	0	0	0	
9	99416267	1	0	0	0	0	0	0	0	
10	4110116	1	0	0	0	0	0	0	0	
10	73983507	1	0	0	0	0	0	0	0	
10	113427868	1	0	0	0	0	0	0	0	
10	116113184	1	0	0	0	0	0	0	0	
10	117439774	1	0	0	0	0	0	0	0	
10	117648172	1	0	0	0	0	0	0	0	
10	123726196	1	0	0	0	0	0	0	0	
11	9291411	1	0	0	0	0	0	0	0	
11	108042218	1	0	0	0	0	0	0	0	
12	7492666	1	0	0	0	0	0	0	0	
12	42406086	1	0	0	0	0	0	0	0	
12	99902917	1	0	0	0	0	0	0	0	
12	113080974	1	0	0	0	0	0	0	0	
12	126702950	1	0	0	0	0	0	0	0	
13	33805290	1	0	0	0	0	0	0	0	
13	36640133	1	0	0	0	0	0	0	0	
13	43590775	1	0	0	0	0	0	0	0	
13	93928856	1	0	0	0	0	0	0	0	
13	102958389	1	0	0	0	0	0	0	0	
14	98150947	1	0	0	0	0	0	0	0	
15	83616251	1	0	0	0	0	0	0	0	
15	90057690	1	0	0	0	0	0	0	0	
15	90105573	1	0	0	0	0	0	0	0	
18	36402506	1	0	0	0	0	0	0	0	
19	4151358	1	0	0	0	0	0	0	0	
19	12662937	1	0	0	0	0	0	0	0	
19	38438387	1	0	0	0	0	0	0	0	
20	12842359	1	0	0	0	0	0	0	0	
20	63286812	1	0	0	0	0	0	0	0	
22	42379487	1	0	0	0	0	0	0	0	
1	160604872	0	1	0	0	0	0	0	0	ins 3bp to snp
12	23425365	0	1	0	0	0	0	0	0	hom del 1bp to het del 1bp
10	128598359	0	1	0	0	0	0	0	0	het del 3bp to hom del 3bp
11	31038074	0	1	0	0	0	0	0	0	ins 4bp to 1bp
1	18474502	0	0	1	0	0	0	0	0	

5	112564495	0	0	1	0	0	0	0	0	del 3bp to 1bp
8	31830302	0	0	1	0	0	0	0	0	TTCC to TTAA
5	155865083	0	0	1	0	0	0	0	0	hom ins 4bp to het ins 4bp
15	82612846	0	0	1	0	0	0	0	0	del 3bp to del 1bp
1	8789282	0	0	0	1	0	0	0	0	del 1bp to >4bp
6	149765979	0	0	0	1	0	0	0	0	ins 1bp to ins 2bp
15	65494646	0	0	0	1	0	0	0	0	hom ins 3bp to het ins 3bp
10	52166430	0	0	0	1	0	0	0	0	het snp to hom snp
1	146859636	0	0	0	0	1	0	0	0	
5	180455518	0	0	0	0	1	0	0	0	het snp to hom snp
17	138086	0	0	0	0	0	1	0	0	G to C
1	161851141	0	0	0	0	0	0	1	0	
3	52843633	0	0	0	0	0	0	0	1	ins 1bp to ins 2bp
4	608640	0	0	0	0	0	0	0	1	hom ins 3bp to hom del 3bp, 2 alt. alleles candidate
4	69524125	0	0	0	0	0	0	0	1	snp to ins 1bp, 2 alt. alleles candidate
6	164316983	0	0	0	0	0	0	0	1	ins 1bp to 2bp
13	23318204	0	0	0	0	0	0	0	1	ins 1bp to snp
18	74007219	0	0	0	0	0	0	0	1	ins 2bp to ins >4bp
1	17964531	0	0	0	0	0	0	0	1	het snp to hom snp
2	139522536	0	0	0	0	0	0	0	1	het snp to hom snp
4	91168578	0	0	0	0	0	0	0	1	het snp to hom snp
4	132108948	0	0	0	0	0	0	0	1	G to T
6	68830955	0	0	0	0	0	0	0	1	
7	21608839	0	0	0	0	0	0	0	1	ins 2bp to ins 3bp
Sum		71	4	5	4	2	1	1	12	

Supplementary Table 5. Reasons for false negative variant calls.

The details of the 100 randomly picked false negative variant calls.

Chromosome	Position	Two alternative alleles	Low complexity	Tandem repeat	Polymer run	Low depth	Very low depth	No depth	No significant reason	Comments
1	17003585	1	0	0	0	0	0	0	0	
1	97883834	1	0	0	0	0	0	0	0	
1	101219815	1	0	0	0	0	0	0	0	
1	164144467	1	0	0	0	0	0	0	0	
1	171651348	1	0	0	0	0	0	0	0	
1	176278303	1	0	0	0	0	0	0	0	
1	183688456	1	0	0	0	0	0	0	0	
2	51032510	1	0	0	0	0	0	0	0	
2	102112311	1	0	0	0	0	0	0	0	
2	118260336	1	0	0	0	0	0	0	0	
2	221277848	1	0	0	0	0	0	0	0	
4	156455599	1	0	0	0	0	0	0	0	
5	129990377	1	0	0	0	0	0	0	0	
6	15733543	1	0	0	0	0	0	0	0	
6	23119952	1	0	0	0	0	0	0	0	
6	25742512	1	0	0	0	0	0	0	0	
6	69436627	1	0	0	0	0	0	0	0	
6	122436195	1	0	0	0	0	0	0	0	
7	11529890	1	0	0	0	0	0	0	0	
7	105201635	1	0	0	0	0	0	0	0	
10	34608534	1	0	0	0	0	0	0	0	
10	113086773	1	0	0	0	0	0	0	0	
11	9375041	1	0	0	0	0	0	0	0	
11	58048677	1	0	0	0	0	0	0	0	
11	69727978	1	0	0	0	0	0	0	0	
12	19651895	1	0	0	0	0	0	0	0	
12	29099347	1	0	0	0	0	0	0	0	
12	75993185	1	0	0	0	0	0	0	0	
13	19860470	1	0	0	0	0	0	0	0	
13	22778831	1	0	0	0	0	0	0	0	
14	60284707	1	0	0	0	0	0	0	0	
14	61688299	1	0	0	0	0	0	0	0	
14	78101794	1	0	0	0	0	0	0	0	

14	84104857	1	0	0	0	0	0	0	0	
14	88301652	1	0	0	0	0	0	0	0	
15	33026410	1	0	0	0	0	0	0	0	
15	52626529	1	0	0	0	0	0	0	0	
15	70600622	1	0	0	0	0	0	0	0	
17	15306089	1	0	0	0	0	0	0	0	
18	63577356	1	0	0	0	0	0	0	0	
19	8162197	1	0	0	0	0	0	0	0	
19	33269330	1	0	0	0	0	0	0	0	
10	123597378	0	1	0	0	0	0	0	0	
18	68026240	0	1	0	0	0	0	0	0	ins 1bp to ins 0bp
3	34279080	0	0	1	0	0	0	0	0	hom ins 4bp to het ins 4bp
3	129412428	0	0	1	0	0	0	0	0	hom del 4bp to het del 4bp
4	89380862	0	0	1	0	0	0	0	0	hom ins 4bp to het ins 4bp
5	91158849	0	0	1	0	0	0	0	0	hom del 4bp to het del 4bp
6	86167385	0	0	1	0	0	0	0	0	del 2bp to del 1bp
6	166439103	0	0	1	0	0	0	0	0	ins 3bp to ins 1bp
10	77619337	0	0	1	0	0	0	0	0	
12	114698176	0	0	1	0	0	0	0	0	het ins 2bp to hom ins 2bp
13	56215838	0	0	1	0	0	0	0	0	hom ins 2bp to het ins 2bp
18	26912372	0	0	1	0	0	0	0	0	ins 4bp to ins 2bp
1	173390750	0	0	0	1	0	0	0	0	ins TT to TC
4	185572414	0	0	0	1	0	0	0	0	ins 1bp to ins 2bp
9	87124801	0	0	0	1	0	0	0	0	insert T to G
12	20982234	0	0	0	1	0	0	0	0	ins 1bp to snp
3	171493464	0	0	0	1	0	0	0	0	del 1bp to ref
4	23299557	0	0	0	1	0	0	0	0	snp to ref
4	143274979	0	0	0	1	0	0	0	0	het ins 1bp to hom ins 1bp
10	90988454	0	0	0	1	0	0	0	0	insert G to A
13	30990346	0	0	0	1	0	0	0	0	del 1bp to snp
13	68427543	0	0	0	1	0	0	0	0	del 3bp ref
1	148674636	0	0	0	0	1	0	0	0	
1	247809552	0	0	0	0	1	0	0	0	het snp to hom snp
3	166825638	0	0	0	0	1	0	0	0	het snp to hom snp
5	79609019	0	0	0	0	1	0	0	0	het snp to hom snp
7	68483896	0	0	0	0	1	0	0	0	snp to ref
7	93174966	0	0	0	0	1	0	0	0	het ins 1bp to hom ins 2bp

9	98851046	0	0	0	0	1	0	0	0	snp to ref
10	46481099	0	0	0	0	1	0	0	0	snp to ref
13	18415324	0	0	0	0	1	0	0	0	snp to ref
22	45054934	0	0	0	0	1	0	0	0	snp to ref
1	120282649	0	0	0	0	0	1	0	0	
3	3952250	0	0	0	0	0	1	0	0	
11	1953662	0	0	0	0	0	1	0	0	
11	2007060	0	0	0	0	0	1	0	0	
15	22200568	0	0	0	0	0	1	0	0	
21	34483345	0	0	0	0	0	1	0	0	
22	15257476	0	0	0	0	0	1	0	0	
11	27601366	0	0	0	0	0	1	0	0	
2	31646165	0	0	0	0	0	0	1	0	del 2bp to ref
3	92647420	0	0	0	0	0	0	1	0	snp to ref
4	21239255	0	0	0	0	0	0	1	0	snp to ref
11	2205559	0	0	0	0	0	0	1	0	snp to ref
4	64922911	0	0	0	0	0	0	1	0	del 4bp to ref
8	61996166	0	0	0	0	0	0	1	0	snp to ref
8	61996194	0	0	0	0	0	0	1	0	snp to ref
10	127321376	0	0	0	0	0	0	0	1	inserted base TG to TT
12	107845650	0	0	0	0	0	0	0	1	ins 1bp to snp
1	109833986	0	0	0	0	0	0	0	1	snp to ref
1	241953921	0	0	0	0	0	0	0	1	snp to ref
4	184253917	0	0	0	0	0	0	0	1	snp to ref
5	113885312	0	0	0	0	0	0	0	1	het ins 1bp to hom ins 1bp
10	53295967	0	0	0	0	0	0	0	1	ins 1bp to ref
11	20578381	0	0	0	0	0	0	0	1	snp to ref
11	90097963	0	0	0	0	0	0	0	1	het del 2bp to hom del 2bp
17	48981858	0	0	0	0	0	0	0	1	snp to ref
19	29196975	0	0	0	0	0	0	0	1	2 alt. alleles candidate
Sum		42	2	10	10	10	8	7	11	

Supplementary Table 6. Performance of variant calls in HG003 on three different models

Three different models include HG001 only, HG002 only and HG001+HG002.

Train using Variants in	Best Variant Quality Cutoff	Overall			SNP			Indel		
		Precision	Recall	F1 Score	Precision	Recall	F1 Score	Precision	Recall	F1 Score
HG001	72	95.61%	90.51%	92.99%	95.61%	90.51%	92.99%	95.61%	90.51%	92.99%
HG002	71	93.38%	88.09%	90.66%	93.38%	88.09%	90.66%	93.38%	88.09%	90.66%
HG001 + HG002	56	95.91%	91.29%	93.54%	95.91%	91.29%	93.54%	95.91%	91.29%	93.54%

Supplementary Table 7. F1-scores of different datasets on different network designs.

Both the original models and slim models were trained on HG001 using the fast training mode.

	Illumina		PacBio		ONT
Models:	HG001	HG002	HG001	HG002	HG001
Original	99.59%	99.50%	95.62%	95.39%	89.34%
Slim	98.77%	98.77%	93.94%	93.49%	87.11%
Degraded	0.82%	0.73%	1.68%	1.90%	2.23%

Supplementary Data

Supplementary Data 1: 3,135 potential novel variants.

Attached as an individual file named “Supplementary Data 1.xlsx”.

Supplementary Note

Unit tests

The unit tests in this section answer the following questions, including 1) what are the characteristics of false positives and false negatives? 2) can lower learning rate and longer training provide better performance? 3) can a model train on truth variants from multiple samples provide better performance? 4) can a higher input data quality improve the variant calling performance? And 5) is the current network design sufficient in terms of learning capacity? We carried out the unit tests by evaluating Clairvoyante's performance on known GIAB truth variant sites to simplify the tests with less latent variables. Please notice that, while the GIAB dataset contains no non-variant sites, the benchmark results shown in this section are biased from the real-life variant calling and are only suitable for comparison among themselves. For clairvoyante's real-life performance, please refer to the two sections in the main text titled "**Call variants at common variant sites**" and "**Genome-wide variant identification**".

We used the submodule *vcfeval* in RTG Tools[1] version 3.7 to benchmark our results and generate three metrics including Precision, Recall, and F1-score. From the number of true positives (TP), false positives (FP), and false negatives (FN), we compute the three metrics as Precision = $TP \div (TP + FP)$, Recall = $TP \div (TP + FN)$, and F1-score = $2TP/(2TP + FN + FP)$. FP are defined as variants existing in the GIAB dataset that also identified as a variant by Clairvoyante, but with discrepant variant type, alternative allele or zygosity. FN are defined as the variants existing in the GIAB dataset but identified as a non-variant by Clairvoyante. F1-score is the harmonic mean of the precision and recall. RTG *vcfeval* also provides the best variant quality cutoff for each dataset, filtering the variants under which can maximize the F1-score.

Characterization of false positives and false negatives

While we have arrived at a highly optimized version of Clairvoyante for the experiments in this paper, it is essential to study the remaining FP and FN variant calls and how they are distributed to support for future improvements. To achieve this, on Illumina data, we have randomly picked 100 FP and 100 FN from the variants called in HG002 using the model trained on HG001 using the fast training mode (stopped at 67-epoch), generated plots on their input and output and manually inspected each one. A summary of the results is shown in **Supplementary Figure 3**. The most significant category of FP and FN variants, accounting for 71 FP and 42 FN, are variants with two or more alternative alleles at the same position. Clairvoyante does not currently support this type of variant, and instead, only one allele will be reported (this limitation is further discussed in the **Main text, Discussion**). Except for 1 FP and 7 FN that have no read coverage at all (because we have downsampled from 300x to 50x), the other 28 FP and 51 FN are errors that Clairvoyante should avoid. Among them, 13 FP and 2 FN failed because of relatively "difficult reference" (low complexity sequence, tandem repeat or homopolymer run), 3 FP and 18 FN because of "lack of evidence" (depth ≤ 20 or even ≤ 3). The results suggest that to improve Clairvoyante further, we should increase the accuracy of the variants in the "difficult reference" regions and increase the sensitivity of the variants "lack of evidence". Noteworthy, the 1 FP Clairvoyante made with no read coverage at all is specific to the "Call variant at only truth variant sites" mode since Clairvoyante will decide on each known site regardless of covered or not. This type of FP could be easily eliminated by filtering the variants with zero depth, but we have retained it in our study to show a complete spectrum of errors Clairvoyante has made. More details for

each FP and FN are shown in **Supplementary Tables 4, and 5** and the plots are available online at http://www.bio8.cs.hku.hk/gallery/fpfnPlots.zip.

Can lower learning rate and longer training provide better performance?

The benchmarking results on the three models stopping at different learning rates allow us to study whether lower learning rate can provide better results and derive how much training is enough. For ONT, both from 999-epoch to 1499-epoch and from 1499-epoch to 1999-epoch, significant improvements were observed (**Table 4**). However, in PacBio (**Table 3**), from 1499-epoch to 1999-epoch, the F1-Score remained the same or increased very slightly (95.78% to 95.78%, 95.99% to 96.03%) when both variant calling and model training is using the same sample, but decreased slightly (94.91% to 94.87%, 94.47% to 94.34%) when using different samples. The results suggest that Clairvoyante was slightly overfitting the training data with a too low learning rate. The same behavior, although very slight, is also observed in Illumina data (**Table 2**). Thus, we suggest the Clairvoyante users to 1) stop at a higher learning rate for less noisy data; 2) train multiple samples stopping at different learning rates and select the best through performance evaluation; or 3) use a model trained on truth variants from multiple samples.

Can a model train on truth variants from multiple samples provide better performance?

Intuitively, a model trained on truth variants from two or more samples should perform better than those trained on just a single sample, provided that the truth variants from different samples have similar high quality. The model might even be more versatile if the characteristics of input, such as average depth, differ between samples. To verify our hypothesis, we benchmarked the known GIAB truth variants called in HG003 (**Supplementary Note, Data Source, PacBio Data**) on three different models trained on 1) HG001; 2) HG002, and; 3) HG001+HG002. All three models were trained for 1000 epochs at learning rate $1e^{-3}$, then another 500 epochs at learning rate $1e^{-4}$. Noteworthy, the time used for training the HG001+HG002 model doubled, as it doubled the number of true variants and paired non-variants. If our hypothesis is correct, the variant calling performance should increase for HG003 when using the HG001+HG002 model than the HG001 model or the HG002 model. The results are shown in **Supplementary Table 6**. Using the HG001+HG002 model, the F1-score is 0.55% higher than using HG001 only and 2.88% higher than using the HG002 only. We conclude that using multiple samples for model training can increase the performance of Clairvoyante, although we expect marginal improvement gains when using more than a few samples.

Can a higher input data quality improve the variant calling performance?

In **Table 4**, we used the 'rel3' ONT dataset generated by the Nanopore WGS consortium. Very recently, the consortium released an augmented dataset labeled 'rel5' (see **Supplementary Note, Data Source, Oxford Nanopore Data**). The 'rel5' data are a merge of NA12878 DNA sequencing data from 'rel3' (regular sequencing protocols, about 30x) and 'rel4' (ultra-read set, 7.7x extra), recalled with the latest base-caller. Thus, we expect to see improved performance, given that the input data quality limited the performance of Clairvoyante on ONT. We trained a model on 'rel5' for 999 epochs at learning rate $1e^{-3}$.

Compare to ‘rel3’, on known GIAB truth variants, the precision improved from 94.07% to 97.21%, the recall improved from 85.87% to 88.80%, and the F1-Score improved from 89.79% to 92.81%. Thus, the results reflect our intuition that Clairvoyante’s performance on ONT data is limited by the input data quality and thus will improve over time as the technology, base-calling mature, and more data become available.

Network topology and capacity evaluation

In the previous subsection, we have shown Clairvoyante’s capacity to perform better on noisy PacBio and ONT data when trained with more data of higher quality. We next evaluated the performance by considering a “slim version” of Clairvoyante with smaller capacity that could potentially improve computational requirements. With the slim version, we expect to see a greater performance in higher quality Illumina data than noisy data like ONT and PacBio data as the classification problem is easier with less noisy data. The slim version includes 165k parameters, which is about ten times fewer than the original version. Instead of isometrically scaling down the original network, we evaluated several different designs resulting in some network components with significantly reduced runtime than others or even reducing the parameters by ten times while still achieving the best runtime and F1-Score possible.

Our final slim network design removes the pooling between convolutional layers, slightly enlarged the kernel size in convolution and reduced the number of nodes in the two fully-connected layers by ten times. We trained models using the fast training mode on HG001 and benchmarked the Illumina, PacBio and ONT data on both HG001 and HG002. The benchmarking results on known GIAB truth variants are shown in **Supplementary Table 7**. As expected, the F1-scores degraded least in the Illumina datasets (0.82% and 0.73%) and degraded most in the ONT dataset (2.23%), with PacBio in the middle (1.68% and 1.90%). The slim version is available as a part of the Clairvoyante toolset and can be enabled with option ‘--slim.’

Pseudo code for generating the input

```
Input: p, genome position of a candidate variant, 1-based
       r, reference genome
       a, a set of elements representing the read alignments covering p at bp level,
          each element contains four members:
          1) the matched reference position (refpos), 1-based,
          2) the offset of this bp within its insertion pattern (insoffset),
             1-based (only applicable if this bp is an inserted base),
          3) the bp 'A' or 'C' or 'G' or 'T' or the gap '-' in r at refpos (refbase),
          4) the bp or the gap in the corresponding aligned read at readpos (readbase).

Output: A matrix x of order 33 by 4 by 4

h.insert('A', 0)
h.insert('C', 1)
h.insert('G', 2)
h.insert('T', 3)

create a zero-filled matrix x of size (33 x 4 x 4)

for each item in a do:
    /* Skip the Ns and IUPAC nucleotides in reference */
    if a[item].refbase not one of "ACGT-" then:
        continue
    /* Skip the Ns in read */
    if a[item].readbase not one of "ACGT-" then:
        continue
    /* Center the candidate variant in the first dimension (33) of x */
    offset <- refpos - p + 16
    /* Skip if beyond scope */
    if offset < 0 or offset > 32 then:
        continue
    /* Translate refbase and readbase into index */
    refbaseIndex <- h.get(a[item].refbase)
    readbaseIndex <- h.get(a[item].readbase)
    /* If not a deleted base */
    if a[item].readbase ≠ '-' then:
        /* If not a inserted base either, i.e. a ref or a SNP */
        if a[item].refbase ≠ '-' then:
            increase x[offset][refbaseIndex][0] by 1
            increase x[offset][readbaseIndex][1] by 1
            increase x[offset][refbaseIndex][2] by 1
            increase x[offset][readbaseIndex][3] by 1
        /* If is a inserted base */
        if a[item].refbase = '-' then:
            newOffset <- min(offset + a[item].insoffset, 32)
            increase x[newOffset][readbaseIndex][1] by 1
    /* If is a deleted base */
    if a[item].readbase = '-' then:
        increase x[offset][refbaseIndex][2] by 1

for i = 0 to 32 do:
    for j = 0 to 3 do:
        x[i][j][1] <- x[i][j][1] - x[i][j][0]
        x[i][j][2] <- x[i][j][2] - x[i][j][0]
        x[i][j][3] <- x[i][j][3] - x[i][j][0]

return x
```

Data Sources

Truth Variants (Genome in a Bottle dataset version 3.3.2)

HG001 (NA12878), GRCh38

ftp://ftp-trace.ncbi.nlm.nih.gov/giab/ftp/release/NA12878_HG001/NISTv3.3.2/GRCh38

HG001 (NA12878), GRCh37

ftp://ftp-trace.ncbi.nlm.nih.gov/giab/ftp/release/NA12878_HG001/NISTv3.3.2/GRCh37

HG002 (NA24385), GRCh38

ftp://ftp-trace.ncbi.nlm.nih.gov/giab/ftp/release/AshkenazimTrio/HG002_NA24385_son/NISTv3.3.2/GRCh38

HG002 (NA24385), GRCh37

ftp://ftp-trace.ncbi.nlm.nih.gov/giab/ftp/release/AshkenazimTrio/HG002_NA24385_son/NISTv3.3.2/GRCh37

HG003 (NA24149), GRCh37

ftp://ftp-trace.ncbi.nlm.nih.gov/giab/ftp/release/AshkenazimTrio/HG003_NA24149_father/NISTv3.3.2/GRCh37/

Common Variants (1000 genome project, phase 3)

HG001 (NA12878), GRCh37

http://ftp.1000genomes.ebi.ac.uk/vol1/ftp/release/20130502/

HG001 (NA12878), GRCh38

http://ftp.1000genomes.ebi.ac.uk/vol1/ftp/release/20130502/supporting/GRCh38_positions/

Illumina Data

HG001 (NA12878), GRCh38

ftp://ftp-trace.ncbi.nlm.nih.gov/giab/ftp/data/NA12878/NIST_NA12878_HG001_HiSeq_300x/NHGRI_Illumina300X_novoalign_bams/HG001.GRCh38_full_plus_hs38d1_analysis_set_minus_alts.300x.bam

# Further down-sampled to 50x using command 'samtools view -s 0.166666' [2].

HG002 (NA24385), GRCh38

ftp://ftp-trace.ncbi.nlm.nih.gov/giab/ftp/data/AshkenazimTrio/HG002_NA24385_son/NIST_Illumina_2x250bps/novoalign_bams/HG002.GRCh38.2x250.bam

# Further down-sampled to 50x using command 'samtools view -s 0.166666'.

The reference genome

ftp://ftp.ncbi.nlm.nih.gov/genomes/all/GCA/000/001/405/GCA_000001405.15_GRCh38/seqs_for_alignment_pipelines.ucsc_ids/GCA_000001405.15_GRCh38_no_alt_plus_hs38d1_analysis_set.fna.gz

Pacific Bioscience (PacBio) Data

HG001 (NA12878), GRCh37

ftp://ftp-trace.ncbi.nlm.nih.gov/giab/ftp/data/NA12878/NA12878_PacBio_MtSinai/sorted_final_merged.bam

# Reads were extracted from the bam file and realigned to reference hs37d5 using aligner NGMLR v0.2.3 [3].

HG002 (NA24385), GRCh37

ftp://ftp-trace.ncbi.nlm.nih.gov/giab/ftp/data/AshkenazimTrio/HG002_NA24385_son/PacBio_MtSinai_NIST/Baylor_NGMLR_bam_GRCh37/all_reads.fa.giab_h002_ngmlr-0.2.3_mapped.bam

HG003 (NA24149), GRCh37

ftp://ftp-trace.ncbi.nlm.nih.gov/giab/ftp/data/AshkenazimTrio/HG003_NA24149_father/PacBio_MtSinai_NIST/Baylor_NGMLR_bam_GRCh37/all_reads.fa.giab_h003_ngmlr-0.2.3_mapped.bam

The reference genome

ftp://ftp.1000genomes.ebi.ac.uk/vol1/ftp/technical/reference/phase2_reference_assembly_sequence/hs37d5.fa.gz

Oxford Nanopore (ONT) Data

HG001 (NA12878) rel3, GRCh37

https://github.com/nanopore-wgs-consortium/NA12878/

HG001 (NA12878) rel5, GRCh37

https://github.com/nanopore-wgs-consortium/NA12878/blob/master/rel5.md

# The alignments were provided by Fritz Sedlazeck through personal communication. Reference genome hs37d5 and aligner NGMLR v0.2.3 [3] were used for generating the alignments. In the GitHub, we have provided models trained on the rel3 data instead of rel5, because rel5 was base-called using the latest base-caller Albacore 2.1, which is too new and has yet accumulated enough evidence on its reliability. We will keep track the rel5 release and provide models trained on rel5 later.

The reference genome

ftp://ftp.1000genomes.ebi.ac.uk/vol1/ftp/technical/reference/phase2_reference_assembly_sequence/hs37d5.fa.gz

Call Variants at Common Variant Sites

Commands

Pre-requisitions

The 1kgp3.afcutoff5.vcf.gz file was generated from the VCF file downloaded from the 1000 genome project website, with variants with global allele frequency (AF) <5% filtered and pre-processed with methods in the section Evaluation, Commands, Normalize the genotype in GT tag.
Read alignments: alignment.bam
The corresponding reference genome “genome.fa” and index “genome.fa.fai”
A Clairvoyante model including three files: model.index, model.meta, model.data-00000-of-00001.
Install Pypy version ≥5.01.
Install Tensorflow version ≥1.0.
Please notice that some reference genome uses “chr” as the prefix for chromosome names and some doesn’t. Please change the “--ctgName” option in the subsections accordingly.

--------------------------------Clairvoyante (the complicated but illustrative way)

Extract the details of Common variant sites

```
for i in {1..22} X Y; do printf "pypy dataPrepScripts/GetTruth.py --vcf_fn 1kgp3.afcutoff5.vcf.gz --var_fn var_chr$i --ctgName chr$i &\n"; done > run.sh
sh run.sh
```

# The commands in the run.sh script can be run in parallel

Generate read alignment arrays for common variants

```
for i in {1..22} X Y; do printf "pypy dataPrepScripts/CreateTensor.py --bam_fn alignment.bam --can_fn var_chr$i --ctgName chr$i --ref_fn genome.fa --tensor_fn array_chr$i &\n"; done > run.sh
sh run.sh
```

# The commands in the run.sh script can be run in parallel

Concatenate the arrays

```
for i in {1..22} X Y; do cat array_chr$i; done > array_all
```

Call variants at common variant sites

```
python clairvoyante/callVar.py --thread 28 --chkpnt_fn model --tensor_fn array_all --call_fn calls.vcf
```

Remove insertions and deletions >4bp from the variant calls

```
cat calls.vcf | perl -ane 'if(/^#/){print}else{if(length($F[3])==1 && length($F[4])==1){print}elsif(length($F[3])==1 && length($F[4])<=5){print}elsif(length($F[3])<=5 && length($F[4])==1){print}}' | grep -v \<INS | grep -v \<DEL | bgziptabix  calls.withOutSV.vcf.gz
```

--------------------------------Clairvoyante End

--------------------------------Clairvoyante Extension (a way simpler than what we have used in our study)

A simpler and compact way

#Please note that the next command runs callVarBam.py on each chromosome one by one. To speed up, one can print out the commands and run them in parallel subject to the number of available CPU cores.

for i in {1..22} X Y; do python clairvoyante/callVarBam.py --vcf_fn 1kgp3.afcutoff5.vcf.gz --chkpnt_fn model --call_fn output.vcf --ref_fn genome.fa --sampleName HG001 --bam_fn alignment.bam --ctgName chr$i; done

vcfcat *.chr{1..22}.vcf *.chr{X,Y}.vcf | perl -ane 'if(/^#/){print}else{if(length($F[3])==1 && length($F[4])==1){print}elsif(length($F[3])==1 && length($F[4])<=5){print}elsif(length($F[3])<=5 && length($F[4])==1){print}}' | grep -v \<INS | grep -v \<DEL | bgziptabix all.withoutSV.vcf.gz

--------------------------------Clairvoyante Extension End

--------------------------------GATK

samtools addreplacerg -r 'ID:HG001\tPL:ILLUMINA\tSM:HG001' alignment.bam > alignment.rg.bam

java -Djava.io.tmpdir=/dev/shm -Xmx50G -jar GenomeAnalysisTK-3.7.jar -T UnifiedGenotyper -I alignment.rg.bam -o unifiedGenotyper.vcf.gz -R genome.fa -nt 28 --defaultBaseQualities 40 -glm BOTH

# using GATK UnifiedGenotyper for Indel calling (using -glm BOTH) is only applicable on Illumina data. Setting -glm BOTH on Single Molecule Sequencing data will take weeks to finish running on a genome.

java -Djava.io.tmpdir=/dev/shm -Xmx50G -jar GenomeAnalysisTK-3.7.jar -T HaplotypeCaller -I alignment.rg.bam -o haplotypeCaller.vcf.gz -R genome.fa -nct 28 --defaultBaseQualities 40

--------------------------------GATK End

-------------------------------- PacBio GenomicConsensus v5.1

PacBio implemented a module named "GenomicConsensus" in there proprietary SMRT Link software suite for variant calling. The module uses an algorithm named "Quiver" to analyze the aligned reads and generate consensus sequences, after which variants will be identified as the differences between the consensus sequences and a target reference genome. The consensus step requires several hundred thousand CPU hours as reported by PacBio in 2014 on a 54x hydatidiform mole sample named CHM1 (GenBank assembly accession GCA_001007805.1). Such a high computational cost might be reasonable for a genome assembly project but is not affordable by most of the people targeting simpler tasks like variant calling. We tried running the latest version of SMRT Link software suite (v5.1) in order to call variants in a small genome region for estimating an approximation of accuracy and sensitivity. However, the software suite enforced a non-standard BAM format and rejected the BAM file we downloaded from the Genome In A Bottle repository.

-------------------------------- PacBio GenomicConsensus v5.1 End

-------------------------------- Nanopolish v0.9.0

We chunked the whole genome into 5Mbp segments and run nanopolish on each segment with 14 concurrencies and 2 threads per job.

nanopolish variants -r reads.fq.gz --genotype 1kgp3.afcutoff5.vcf.gz -b alignment.bam --ploidy 2 -g genome.fa -t 2 -o output_prefix

-------------------------------- Nanopolish v0.9.0 End

-------------------------------- Model training

Please refer to the Python Notebook “jupyter_nb/demo.ipynb” or the demo script “dataPrepScripts/PrepDataBeforeDemo.pypy.sh” for the procedures to train a Clairvoyante model.

-------------------------------- Model training End

-------------------------------- Merge multiple samples for training

Please refer to the demo script “dataPrepScripts/CombineMultipleGenomesForTraining.sh” for an illustration on how to combine the training samples from different genomes.

-------------------------------- Merge multiple samples for training End

Benchmarking

Commands

Pre-requisitions

Install rtg-tools-3.7.1.
Install vcflib (https://github.com/vcflib/vcflib).
The baseline.vcf.gz and high-confidence-region.bed files provided by GIAB.
genome.sdf created by “rtg format” with a reference genome file genome.fa.
calls.withOutSV.vcf.gz, variant calls generated by Clairvoyante or by GATK with methods in section “Call Variants at Common Variant Sites, Commands, Remove insertions and deletions >4bp from the variant calls”.

Pre-processing GIAB baseline VCF file

```
gzip -dc baseline.vcf.gz | vcfbreakmulti | bgziptabix baseline.breakmulti.vcf.gz
```

Retain only the highly confident variants in the baseline

```
rtg-tools-3.7.1/rtg vcffilter --include-bed=high-confidence-region.bed -i baseline.breakmulti.vcf.gz -o
baseline.breakmulti.inbed.vcf.gz
```

Remove insertions and deletions >4bp from the baseline

```
gzip -dc baseline.breakmulti.inbed.vcf.gz | perl -ane 'if(/^#/){print}else{if(length($F[3])==1 &&
length($F[4])==1){print}elsif(length($F[3])==1 && length($F[4])<=5){print}elsif(length($F[3])<=5 &&
length($F[4])==1){print}}' | bgziptabix baseline.breakmulti.inbed.withOutSV.vcf.gz
```

Retain only one record for heterozygous alternative

```
gzip -dc baseline.breakmulti.inbed.withOutSV.vcf.gz | perl -ane
'BEGIN{%a=();}{if(/^#/){print}elsif(not defined $a{"$F[0]-$F[1]"}){print;$a{"$F[0]-$F[1]"}=1;}}' |
bgziptabix baseline.breakmulti.inbed.withOutSV.uniq.vcf.gz
```

Normalize the genotype in GT tag

```
gzip -dc baseline.breakmulti.inbed.withOutSV.uniq.vcf.gz | perl -ane 'if(/^#/){print}else{@a=split
":",$F[-1];$a[0]=~s/\./0/;$a[0]=~s/\|/\//;@b=split
"/",$a[0];if($b[0]>$b[1]){$a[0]="$b[1]/$b[0]"}else{$a[0]="$b[0]/$b[1]"};$F[-1]=join ":",@a; print join
"\t", @F;print "\n";}' | bgziptabix baseline.breakmulti.inbed.withOutSV.uniq.normalizeGT.vcf.gz
```

RTG vcfeval

```
rtg-tools-3.7.1/rtg vcfeval -t genome.sdf -e high-confidence-region.bed -b
baseline.breakmulti.inbed.withOutSV.uniq.normalizeGT.vcf.gz -c calls.withOutSV.vcf.gz -o
benchmarkingResultsFolder
```

Call Variants Genome-wide

Commands

Pre-requisitions

The high-confidence-region.bed file provided by GIAB.
Read alignments: alignment.bam
The corresponding reference genome “genome.fa” and index “genome.fa.fai”.
A Clairvoyante model including three files: model.index, model.meta, model.data-00000-of-00001.
Install Pypy version ≥5.01.
Install Tensorflow version ≥1.0.
Please notice that some reference genome uses “chr” as the prefix for chromosome names and some doesn’t. Please change the “--ctgName” option in the subsections accordingly.

--------------------------------Clairvoyante

Generate a BED file that covers the whole genome

```
awk ‘{print $1”\t0\t”$2}’ genome.fa.fai > genome.bed
```

Generate scripts that call variants in genome chucks (with alternative allele frequency cutoff at 0.2, minimum coverage at 4, divide gnome into 10Mbp per chunk, “HG001” as the sample name in VCF, output VCF files with prefix “out”)

```
python clairvoyante/callVarBamParallel.py --chkpnt_fn model --ref_fn genome.fa --bed_fn
genome.bed --bam_fn alignment.bam --sampleName HG001 --output_prefix out --
tensorflowThreads 4 --threshold 0.2 --minCoverage 4 --refChunkSize 10000000  > run.sh
```

Run the scripts in parallel (with 28 concurrency)

```
cat run.sh | awk '{print "\""$0"\""}' | xargs -L1 -P 28 sh -c
```

Concatenate the VCF files and remove insertions and deletions >4bp from the calls

```
perl -ne '/\s+(out.\S+.vcf)\s+/;print "$1\n";' run.sh > output.list

vcfcat `perl -ne 'chomp;print "$_ "' output.list` | perl -ane 'if(/^#/){print}else{if(length($F[3])==1 &&
length($F[4])==1){print}elsif(length($F[3])==1 && length($F[4])<=5){print}elsif(length($F[3])<=5 &&
length($F[4])==1){print}}' | grep -v \<INS | grep -v \<DEL | bgziptabix calls.withOutSV.vcf.gz
```

Retain only the highly confident variants in the calls

```
rtg-tools-3.7.1/rtg vcffilter --include-bed=high-confidence-region.bed -i calls.withOutSV.vcf.gz -o
calls.withOutSV.vcf.gz
```

--------------------------------Clairvoyante End

--------------------------------Vardict

genome.region is a file containing the genome region coordinations at 10Mbp chunk size
For ONT, an additional option “-k 0” is added

```
p=`pwd`;for i in `cat genome.region`; do echo "\"VarDict -G genome.fa -N sampleName -b
alignments.bam -R $i | Rscript teststrandbias.R | var2vcf_valid.pl -N NA12878 > $p/$i.vcf\""; done |
xargs -P 24 -L 1 sh -c
```

--------------------------------Vardict End

--------------------------------LoFreq

For ONT, an additional options “-B -A -a 0.01 -q 0 -Q 0 --no-default-filter” are added
lofreq call-parallel --pp-threads 24 -f genome.fa -o lofreq.vcf alignments.bam

--------------------------------LoFreq End

--------------------------------Freebayes

For ONT, an additional option “--use-best-n-alleles 2” is added
freebayes-parallel <(~fasta_generate_regions.py genome.fa.fai 10000000) 24 -f genome.fa --strict-vcf -= alignments.bam > freebayes.vcf

--------------------------------Freebayes End

--------------------------------DeepVariant

We used DeepVariant version 0.6.1 for benchmarking following guide "Improve DeepVariant for BGISEQ germline variant calling" written by Pi-Chuan Chang available at link https://goo.gl/tg4FWG with specific guidelines for how to run DeepVariant, including 1) model training using transfer-learning and multiple depths, and 2) variant calling.

-------------------------------- DeepVariant End

Supplemental References

1 Cleary, J. G. *et al.* Joint variant and de novo mutation identification on pedigrees from high-throughput sequencing data. *J Comput Biol* **21**, 405-419, doi:10.1089/cmb.2014.0029 (2014).

2 Li, H. *et al.* The Sequence Alignment/Map format and SAMtools. *Bioinformatics* **25**, 2078-2079, doi:10.1093/bioinformatics/btp352 (2009).

3 Sedlazeck, F. J. *et al.* Accurate detection of complex structural variations using single molecule sequencing. *bioRxiv*, doi:10.1101/169557 (2017).
