# Supplementary material for: A multi-task convolutional deep neural network for variant calling in single molecule sequencing
Source: Nat Commun. 2019 Mar 1;10:998. doi: 10.1038/s41467-019-09025-z (PMC6397153; doi:10.1038/s41467-019-09025-z)
Supplement: Supplementary file 3 — Description of Additional Supplementary Files [file 41467_2019_9025_MOESM3_ESM.pdf]

Description of Additional Supplementary Files

Supplementary Data 1:
3,135 potential novel variants.
